# Novel theoretical approach to the GISAXS issue: the Green function formalism using the *q*-Eigenwaves propagating through a twofold rough-surfaced medium

**DOI:** 10.1038/s41598-020-68326-2

**Published:** 2020-07-14

**Authors:** F. N. Chukhovskii, B. S. Roshchin

**Affiliations:** Shubnikov Institute of Crystallography of Federal Scientific Research Centre “Crystallography and Photonics”, Russian Academy of Sciences, Leninsky pr. 59, Moscow, Russia 119333

**Keywords:** Surfaces, interfaces and thin films, X-rays

## Abstract

To describe the 1D and 2D patterns of the grazing-incidence small-angle X-ray scattering (GISAXS) from a rough fractal surface, the novel integral equations for the amplitudes of reflected and transmitted waves are derived. To be specific, the analytical expression for the 2D total intensity distribution $\frac{{dR_{tot} \left( {\theta ,\phi ;\theta_{0} } \right)}}{d\Omega }$ is obtained. The latter represents by itself a superposition of terms related to the GISAXS specular $\frac{{dR_{spec} \left( {\theta ;\theta_{0} } \right)}}{d\Omega }$ and diffuse $\frac{{dR_{dif} \left( {\theta ,\phi ;\theta_{0} } \right)}}{d\Omega }$ patterns, respectively. Hereafter, $\theta$ is the scattering meridian angle, $\phi$ is the scattering azimuth angle; $\theta_{0}$ is the angle of incidence. By using the above analytical expressions, the 1D and 2D GISAXS patterns are numerically calculated. Some new experimental measurements of the specular reflectivity curves *R*_spec_($\theta_{0}$) related to the fused quartz and crystal Si(111) samples have been carried out. Based on the theoretical approach developed, a direct least-squared procedure in a *χ*^2^-fit fashion has been used to determine the corresponding values of the root-mean-square roughness *σ* from the specular GISAXS reflectivity data.

## Introduction

The grazing-incidence small-angle X-ray scattering (GISAXS) is worldwide used as a versatile tool for the non-destructive investigation of nanostructure surfaces, study of solid(liquid)-layered structures, interplanetary atom doping, nanoscale surfaces, optical mirrors and etc.^1–5^.

By using advanced technique of crystal growth, fabricating *new* semiconductor materials is based on the idea that the *exciton Bohr radius* plays an important role in the corresponding exciton binding energy and influences onto their electronic and optical key-properties. Should the electronic device is sized well large than the *exciton Bohr radius*, which is typically a few tens of nanometers, the electronic structure exclusively depends on the material property^3^.

In particular, the exciton size depends on the dimensions of the structure and is rather sensitive to local thickness fluctuations as well. Therefore, the field-surfaced roughness is one of the crucial structural parameters that strongly affects the optical and electronic properties, and is needed in a detailed analysis. As to the field-surfaced roughness studies of the *mesoscopic* lateral structures by applying the conventional GISAXS technique^3^, spot sizes of X-ray beams are typically in the range of a few square millimeters. Due to the small incidence angles, the X-ray beam footprint significantly elongates with typical lengths of several millimeters. Then, the conventional GISAXS technique data are very reliable concerning ensemble statistics, covering over the entire *mesoscopic* length range.

To date, the special GISAXS technique with a nanoscale resolution has been developed^6–8^ (see^9^ as well) to avoiding a parasitic scattering from the surrounding structures that bury the nanoscale target signal. To be specific, in the work^8^, authors have fulfilled the GISAXS measurements for isolated and surrounded grating targets in Si substrates with line lengths from 50 μm down to 4 μm, and successfully interpreted the pure GISAXS data obtained due to the reduced target lengths.

Handling to the standard GISAXS method, it provides quantitative information over the root-mean-square (r.m.s.) roughness σ and roughness correlation length $\ell$. The latter are in the range from a few nanometers for the r.m.s. σ and some micrometers for correlation length $\ell$, respectively (see, e.g.^3,6^).

Nevertheless, even in the case of simple *mesoscopic structure* predominates, no one states that the GISAXS theory that is going out beyond both the distorted-wave Born approximation (DWBA) and the pure perturbation theory based on the parameter $k\sigma \theta_{0} < 1$ seems to be completed ($k = 2\pi /\lambda$, $\lambda$ is the X-ray wavelength, $\theta_{0}$ is the grazing-incidence angle).

There are a lot of the theoretical works concerning the GISAXS issue, amid which the works^10–13^ are widely cited. In the work^11^, the static Debye–Waller factors of the reflected and transmitted GISAXS ***q***_0_-waves coherently scattered have been derived. In the framework of the GISAXS perturbation theory for solving the integral stationary wave equation, authors of the work^12^ have developed the Green function method proposed in^10^. In the work^13^, to solve the GISAXS issue, authors have developed the brilliant DWBA formalism widely used to treating the various GISAXS issues. In the paper^14^ authors carefully have compared both the possibilities of the perturbation theory in the framework of the Green function formalism and the conventional DWBA. They have concluded that the conventional DWBA utilizes some simplified assumptions regarding an unperturbed wave field nearly a rough surface, which can be removed by using the Green function theory. Even without any calculations, one can see the similarity and difference of the above theoretical techniques. Their similarity and difference are the following. Both of them use the same set of conventional Fresnel solutions as the **q**-Eigenfunctions to find out the perturbation theory solutions of the GISAXS issue (cf.^14–17^). At the same time, the major difference each to other consists of the following. The conventional DWBA produces the perturbation theory solutions from the beginning, whereas the Green function formalism generates them at the stage of the rigorous asymptotic equations for reflected and transmitted wave amplitudes^14^. From that, it immediately follows that the range validity of the perturbation theory solutions built in the Green function framework cannot be less but only be more than the conventional DWBA solutions.

In the works^15,16^ the so-called self-consistent wave approach (SCWA) to solve the basic integral Green function wave equation has been suggested and analyzed. In particular, one has shown that the SCWA allows the GISAXS solutions to satisfy the optical theorem in the limit of large correlation lengths, $k\ell \to \infty$^16^. The next, to treat the GISAXS issue based on the Green function formalism, one has suggested the ***q***-Eigenfunction wave field approach when the GISAXS issue solution *in search* represents by itself the direct 2D Fourier transform of the ***q***-Eigenwaves propagating through the twofold homogeneous medium^15–17^.

Despite of a definite progress in the GISAXS theory developing, the above theoretical approaches^15–17^ have been not successive by volume, especially, for large values of the r.m.s. roughness σ.

To date, it becomes clear that the Green function formalism needs to be advanced in a proper way aiming to make the GISAXS theory output to be more transparent from both the mathematical (math) and physical viewpoints. Due to the foregoing, the reformulation of the Green function formalism becomes to be of great interest.

A goal of the present study is to develop the GISAXS theory approach based on the Green function formalism to be modified. In other words, our aim is to highlight the modified Green function formalism differed from ones earlier proposed in^15–17^, and in fact, to reformulate the theory fundamentals in a proper way deriving the novel self-consistent (non-averaged) wave field equations to determine the reflected and transmitted GISAXS wave field.

As it will be shown underneath, reformulation of the Green function formalism allows to shorten the intermediate math calculations in comparison with similar ones in^15–17^, and makes the analytical formulae describing the specular (coherent) and non-specular (incoherent) X-ray scattering to be more transparent and understandable. Following to^17^, we will search the wave field $E\left( {{\mathbf{x}},z} \right)$ propagating through the twofold rough-surfaced medium in the form

1 $$E\left( {{\mathbf{x}},z} \right) = \frac{1}{{\left( {2\pi } \right)^{2} }}\int {d^{2} {\mathbf{q}}} \left\{ \begin{array}{ll} \left( {2\pi } \right)^{2} \delta_{2} \left( {{\mathbf{q}} - {\mathbf{q}}_{0} } \right)e^{{i{\mathbf{q}}_{0} {\mathbf{x}} + ik_{z} (q_{0} )z}} + B\left( {\mathbf{q}} \right)e^{{i{\mathbf{qx}} - ik_{z} \left( q \right)z}} &\quad {\text{ for }}z < h\left( {\mathbf{x}} \right), \hfill \\ C\left( {\mathbf{q}} \right)e^{{i{\mathbf{qx}} + i\kappa_{z} \left( q \right)z}} &\quad {\text{for }}z > h\left( {\mathbf{x}} \right) \hfill \\ \end{array} \right\},$$

where *h*(**x**) being the surface height at the 2D planar point coordinate **x**; the average <*h*(**x**)> is assumed to equal zero. $\delta_{2} \left( {{\mathbf{q}} - {\mathbf{q}}_{0} } \right)$ is the 2D delta function related to the incident X-ray plane wave

$$E_{inc} \left( {\mathbf{r}} \right) = e^{{i{\mathbf{q}}_{0} \cdot {\mathbf{x}} + ik_{z} (q_{0} )z}} `{.}$$

Hereafter, the reflected and transmitted amplitudes, *B*(**q**) and *C*(**q**), are the inverse 2D **q**-Fourier transforms of Eigenwavefield (1), ($k_{z} (q)$ = (*k*^2 ^− *q*^2^)^1/2^).

Furthermore, to evaluate the specular (coherent) and non-specular (incoherent) GISAXS scattering, the Gaussian ensemble-statistics model of a randomly rough fractal surface^17–20^ is assumed in terms of the r.m.s. roughness $\sigma = \sqrt {\left\langle {h\left( {\mathbf{x}} \right)^{2} } \right\rangle }$, the averaged roughness <*h*(**x**)> is assumed to be equal to zero and the two-point (height-height) cumulant correlation function ${\text{K}}_{2} \left( x \right) = \frac{{h\left( {x/\ell } \right)^{h} }}{{2^{h - 1} \Gamma \left( {h + 1} \right)}}K_{ - h} \left( {x/\ell } \right),$ where $K_{ - h} \left( {x/\ell } \right)$ is the modified Bessel function of the second kind, $x \equiv \left| {{\mathbf{x}}_{1} - {\mathbf{x}}_{2} } \right|$; $\ell$ is the correlation length, *h* is the fractal-surface model index (FSMI).

In the further study, the power spectrum density function $PSD_{2D} \left( q \right)$-function defined as $PSD_{2D} \left( q \right) = 4\pi h\ell^{2} /\left( {1 + \left( {q\ell } \right)^{2} } \right)^{1 + h}$ is exploited. It is nothing else the ***q***-Fourier transform of the two-point cumulant correlation function ${\text{K}}_{2} \left( x \right)$, where $q = \left| {\mathbf{q}} \right|$, the 2D vector **q** is perpendicular to the unit vector **n** normal to the plane *z* = 0. Notice that in the case of *h* = 1/2, the two-point cumulant correlation function ${\text{K}}_{2} \left( x \right)$ coincides with the exponential function $e^{ - x/\ell }$^13^.

Hereafter, χ is the complex electric susceptibility; χ = Reχ + *i*Imχ, Reχ < 0 and Imχ > 0; *θ*_cr_ = (− Reχ)^1/2^ is the critical angle. For instance, in the case of the incident *CuK*_α1_ radiation (the wavelength λ = 0.154 nm). Accordingly, for the fused quartz and Si(111) samples there are χ and *θ*_cr_ equal to: − 14.232·10^–6^ + *i*0.18281·10^–6^, 3.77253·10^–3^; and − 15.127·10^–6^ + *i*0.34955·10^–6^, 3.88934·10^–3^, respectively.

The present study is exposed as follows.

In “Theoretical fundamentals. The modified Green function formalism” section, formulary of the modified Green function formalism is described in details. In opposite to the conventional Green function formalism^12–17^, the artificial plane interface at coordinate *z* = *T*, |*T*|> > σ, is now introduced to define the conventional Fresnel solutions in terms of the ***q***-Eigenfunction waves propagating in the *direct* and *mirror-reversed* scattering geometry. To derive the integral self-consistent (non-averaged) wave field equations for determining the reflected and transmitted wave amplitudes *B*(**q**) and *C*(**q**), the auxiliary parameter *T* is chosen as *T* < 0 and *T* > 0, respectively, and besides, it should be |*T*|> > σ in order to eliminate *T*-interfering effects due to high values of the random roughness field *h*(**x**).

In “Self-consistence Integral equations for probing the GISAXS issue” section, as a result of the modified Green function framework, the two separate self-consistent wave field equations have been derived for the wave field amplitudes *B*(**q**) and *C*(**q**) no linked each to other by using (*z → *$\mp \infty$)-asymptotic of integral equation that governs the wave field (1) *in search*.

In “Zero- and first-order perturbation theory solutions of the GISAXS problem” section, in the scope of the averaged roughness approximation, analytical expressions for amplitude *B*(**q**) ≈ *B*_0_(**q**) + *B*_1_(**q**), which correspond both the specular, *B*_0_(**q**), and diffuse, *B*_1_(**q**), scattering channels from a randomly rough surface are found out. Concerning the specular amplitude *B*_0_(**q**) and non-specular amplitude *B*_1_(**q**), the corresponding analytical expressions for static specular and non-specular scattering factors are derived.

**Figure 1:**
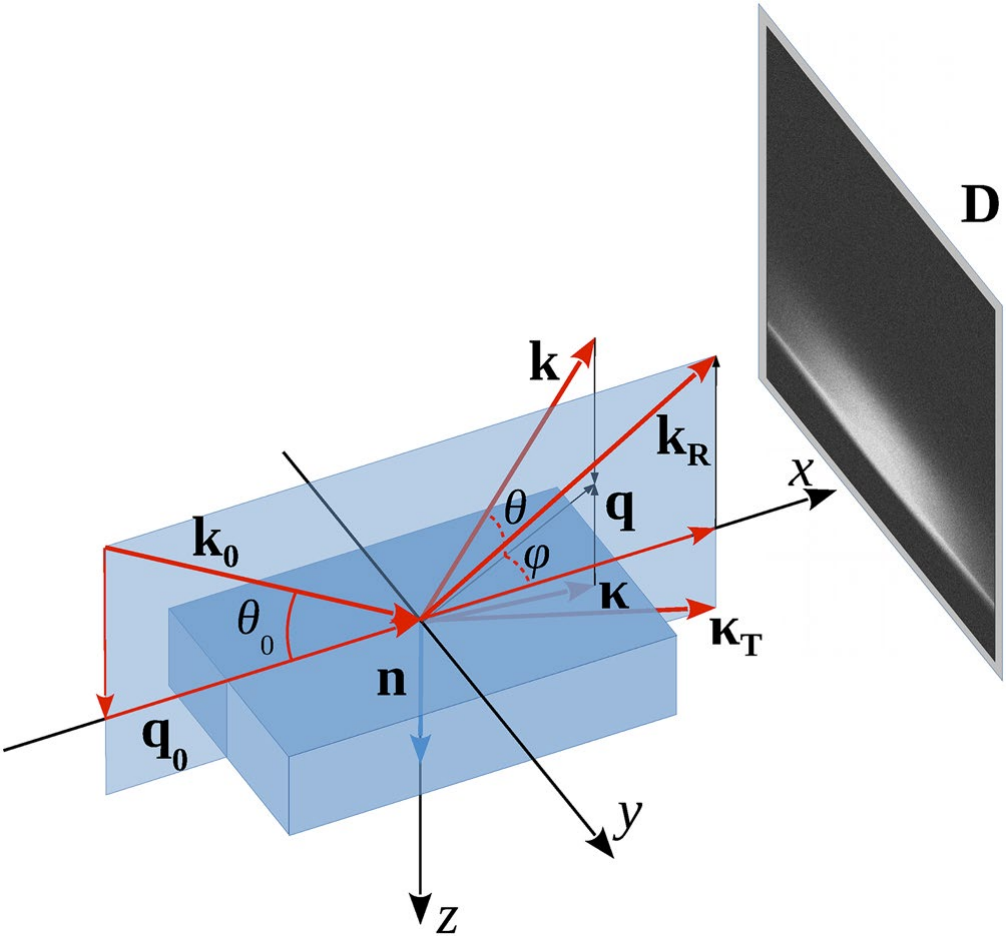
GISAXS layout. k_0_ = **q**_0_ + *k*_z_**n** is the incident wavevector; ***k***_R_ = **q**_0_ − *k*_z_ **n** and ***κ***_T_ = ***q***_0_ + *κ*_z_ **n** are the wavevectors of the specularly reflected and transmitted waves. ***k***_R_ = ***q*** − *k*_z_(q)**n** and ***κ***_T_ = ***q*** + *κ*_z_(*q*)**n** are the wavevectors of the diffuse reflected and transmitted waves. **n** is the unit vector along the *z*-direction perpendicular to the flat surface *z* = 0. Detector D is the 2D CCD camera.

Based on the Gaussian ensemble-statistics, the analytical expressions for the 2D non-specular intensity distribution $\frac{{dR_{dif} }}{d\Omega }\left( {\theta ,\phi ;\theta_{0} } \right)$ and 1D diffuse scattering indicatrix (DSI) $\frac{{dR_{dif} }}{d\theta }\left( {\theta ;\theta_{0} } \right)$ are derived. The DSI $\frac{{dR_{dif} }}{d\theta }\left( {\theta ;\theta_{0} } \right)$ is defined as $\cos \theta \int {d\phi \frac{{dR_{dif} }}{d\Omega }\left( {\theta ,\phi ;\theta_{0} } \right)}$. $\frac{{dR_{dif} }}{d\Omega }\left( {\theta ,\phi ;\theta_{0} } \right)$ is the 2D intensity distribution (2D scan) in the angular range $\left\{ {\theta ,\phi } \right\}$ concerning the fixed grazing-incidence angle $\theta_{0}$, and $\theta$ and $\phi$ are the polar and azimuth angles of the reflected GISAXS beam (see the GISAXS schematic in Fig. 1).

In “Numerical run-through for providing the GISAXS patterns: results and discussion” section, keeping in mind the experimental GISAXS data scans, the computer simulations of the scans $R_{spe} \left( {\theta_{0} } \right)$, $\frac{{dR_{dif} }}{d\Omega }\left( {\theta ,\phi } \right)$ and the DSI $\frac{{dR_{dif} }}{d\theta }\left( \theta \right)$ are presented for various parameters $\left\{ {k\sigma , \, k\ell , \, \theta_{0} /\theta_{cr} } \right\}$ and the FSMI *h*. Herein, in the case of the DSI $\frac{{dR_{dif} }}{d\theta }\left( {\theta ;\theta_{0} } \right)$ the grazing incident angle *θ*_0_ is assumed to be fixed and omitted for simplicity.

In “Concluding remarks” section, some results of the specular GISAXS measurements for the fused quartz and crystal Si(111) samples are presented. The corresponding values of the r.m.s. roughness *σ* are determined by applying standard least-square procedure in a χ^2^-fashion.

## Theoretical fundamentals. The modified Green function formalism

In accordance with the basic idea of the Green function formalism, the integral wave equation can be cast in the form

2 $$E\left( {\mathbf{r}} \right) = E_{0} \left( {\mathbf{r}} \right) - k^{2} \int {d^{3} {\mathbf{r}}_{1} \mathsf{G}\left( {{\mathbf{r}},{\mathbf{r}}_{1} } \right)\Delta \chi \left( {{\mathbf{r}}_{1} } \right)E\left( {{\mathbf{r}}_{1} } \right)} ,$$

where one chooses the electric susceptibility deviation $\Delta \chi \left( {\mathbf{r}} \right)$ as follows (cf.^9,14^)

3 $$\Delta \chi \left( {\mathbf{r}} \right) = \chi \left[ {\Theta \left( {z - h\left( {\mathbf{x}} \right)} \right) -\Theta \left( {z - T} \right)} \right].$$

The Green (a point-source) function $\mathsf{G}\left( {{\mathbf{r}},{\mathbf{r}}_{1} } \right)$ is defined as

4 $$\mathsf{G}\left( {{\mathbf{r}},{\mathbf{r}}_{1} } \right) = - i\left( {4\pi } \right)^{ - 2} \int {d^{2} {\mathbf{q}}\left( {k_{z}^{ - 1} \left( q \right) + \kappa_{z}^{ - 1} \left( q \right)} \right)} \exp \left[ {i{\mathbf{q}}\left( {{\mathbf{x}} - {\mathbf{x}}^{{\prime}} } \right)} \right]\left\{ \begin{array}{ll} y_{2} \left( {z,q} \right)y_{1} \left( {z_{1} ,q} \right), &\quad z \le z_{1} {, } \hfill \\ y_{1} \left( {z,q} \right)y_{2} \left( {z_{1} ,q} \right),&\quad z \ge z_{1} \hfill \\ \end{array} \right.$$

for the twofold medium with the step-like abrupt electric susceptibility: *χ*(**r**) = *χ*Θ(*z* − *T*). Θ(*z* − *Τ*) = 1 for z > *Τ* and Θ(*z* − *Τ*) = 0 for z < *Τ*, respectively; the lateral 2D vector $\left( {{\mathbf{x}} - {\mathbf{x}}^{{\prime}} } \right)$ is lying within the reference plane *z* = 0.

Functions *y*_1_(*z*, *q*) and *y*_2_(*z*, *q*) are the two linearly independent Eigenfunctions of the differential wave equation over the coordinate *z*

5 $${\raise0.7ex\hbox{${d^{2} y}$} \!\mathord{\left/ {\vphantom {{d^{2} y} {dz^{2} }}}\right.\kern-\nulldelimiterspace} \!\lower0.7ex\hbox{${dz^{2} }$}} + \left[ {k^{2} \left( {1 + \chi\Theta \left( {z - {\rm T}} \right)} \right) - q^{2} } \right]y = 0.$$

Noteworthy is the fact that in the Green function method the value of z-coordinate *T* may be arbitrary to some extent. In our case, we choose the value of |*T*| >> σ and sign of z-coordinate *T* being either *T* < 0 or *T* > 0 depending on a goal-problem-setting.

Accordingly, in our case, in the *direct* and *mirror-reversed* GISAXS geometry, solutions of Eq. (5) represent by themselves the conventional Fresnel functions for the step-like abrupt electric susceptibility *χ*Θ(*z* − *T*) as follows

6 $$\begin{aligned} & y_{1} \left( {z,q} \right) = \left\{ \begin{array}{ll} \left( {e^{{ik_{z} \left( q \right)z}} + {\text{R}}_{1} \left( q \right)e^{{ - ik_{z} \left( q \right)z + 2ik_{z} \left( q \right)T}} } \right)&\quad {\text{ for }}z \le {\rm T}, \, \hfill \\ {\text{T}}_{1} \left( q \right)e^{{i\kappa_{z} \left( q \right)z + i\left( {k_{z} \left( q \right) - \kappa_{z} \left( q \right)} \right)T}}&\quad {\text{ for }}z \ge {\rm T} \hfill \\ \end{array} \right. \, , \hfill \\ & y_{2} \left( {z,q} \right) = \left\{ \begin{array}{ll} {\text{T}}_{2} \left( q \right)e^{{ - ik_{z} \left( q \right)z}}&\quad {\text{ for }}z \, \le \, {\rm T}, \hfill \\ e^{{ - i\kappa_{z} \left( q \right)\left( {z - T} \right)}} + {\text{R}}_{2} \left( q \right)e^{{i\kappa_{z} \left( q \right)z - i\left( {k_{z} \left( q \right) + \kappa_{z} \left( q \right)} \right){\rm T}}}&\quad {\text{ for }}z \ge \, {\rm T} \hfill \\ \end{array} \right. \, ,\hfill \\ \end{aligned}$$

respectively.

The reflection R_1_(q), R_2_(q), and transmission T_1_(q), T_2_(q) coefficients take the conventional Fresnel form

7 $$\begin{aligned} & {\text{R}}_{1} \left( q \right) = \frac{{k_{z} \left( q \right) - \kappa_{z} \left( q \right)}}{{k_{z} \left( q \right) + \kappa_{z} \left( q \right)}}, \quad {\text{R}}_{2} \left( q \right) = \frac{{\kappa_{z} \left( q \right) - k_{z} \left( q \right)}}{{k_{z} \left( q \right) + \kappa_{z} \left( q \right)}}, \hfill \\ & {\text{T}}_{1} \left( q \right) = \frac{{2k_{z} \left( q \right)}}{{k_{z} \left( q \right) + \kappa_{z} \left( q \right)}},\quad {\text{ T}}_{2} \left( q \right) = \frac{{2\kappa_{z} \left( q \right)}}{{k_{z} \left( q \right) + \kappa_{z} \left( q \right)}}, \, \hfill \\ \end{aligned}$$

where the *z*-components $k_{z} \left( q \right)$ and $\kappa_{z} \left( q \right)$ in Eqs. (6)–(7) are equal to

8 $$k_{z} \left( q \right) = \sqrt {k^{{2}} - q^{2} } , \quad \kappa_{{\text{z}}} \left( q \right) = \sqrt {\kappa^{2} - q^{2} } ,$$

they relate to the same vector **q** parallel to the plane *z* = 0, *κ*^2^ = *k*^2^(1 + *χ*).

The free term *E*_0_(**r**) in the right-hand side of integral wave Eq. (2) takes the evident form

9 $$E_{0} ({\mathbf{r}}) = \exp \left[ {i{\mathbf{q}}_{0} {\mathbf{x}}} \right]y_{1} \left( {z,q_{0} } \right){.}$$

Hereafter, the following notations are introduced

9a $$k_{z} \left( {q_{0} } \right) \equiv k_{z} , \quad \kappa_{{\text{z}}} \left( {q_{0} } \right) \equiv \kappa_{{\text{z}}} ,\quad {\text{ T}}_{{1, 2}} (q_{0} ) \equiv {\text{T}}_{{1, 2}} ,\quad {\text{ R}}_{{1, 2}} (q_{0} ) \equiv {\text{R}}_{{1, 2}} .$$

As it is seen from Eqs. (2), (3), the integration range over variable *z*1 equal to [Θ(*z*_1_*-T*) − Θ(*z*_1_* − h*(**x**))] determines the behavior of the reflected and transmitted ***q***-Eigenwaves *in search*.

## Self-consistence Integral equations for probing the GISAXS issue

From Eq. (2) it immediately follows that in a limit of the *z*-coordinate tended to $\mp \infty$, the asymptotic GISAXS equations can be cast in the form^17^

10 $$\begin{aligned} & E_{{\text{R}}} \left( {{\mathbf{x}},z} \right)\left| {_{z \to - \infty } } \right. = {\text{R}}_{1} e^{{i{\mathbf{q}}_{0} \cdot {\mathbf{x}} - ik_{z} z + 2ik_{z} T}} \hfill \\ &\quad +\frac{i}{2\pi }\int {\frac{{d^{2} {\mathbf{q}}}}{{k_{z} \left( q \right)}}e^{{i{\mathbf{q}} \cdot {\mathbf{x}} - ik_{z} \left( q \right)z}} A_{{\text{R}}} \left( {q,q_{0} } \right)} , \\ & E_{{\text{T}}} \left( {{\mathbf{x}},z} \right)\left| {_{z \to \infty } } \right. = {\text{T}}_{1} e^{{i{\mathbf{q}}_{0} \cdot {\mathbf{x}} + i\kappa_{z} z + i\left( {k_{z} - \kappa_{z} } \right){\rm T}}} \hfill \\ &\quad+ \frac{i}{2\pi }\int {\frac{{d^{2} {\mathbf{q}}}}{{\kappa_{z} \left( q \right)}}e^{{i{\mathbf{q}} \cdot {\mathbf{x}} + i\kappa_{z} \left( q \right)z + i\left( {k_{z} \left( q \right) - \kappa_{z} \left( q \right)} \right){\rm T}}} A_{{\text{T}}} \left( {q,q_{0} } \right)} , \hfill \\ \end{aligned}$$

whereas the scattering amplitudes $A_{{\text{R}}} \left( {q,q_{0} } \right)$ and $A_{{\text{T}}} \left( {q,q_{0} } \right)$ are nothing else the inverse **q**-Fourier transforms of the corresponding integrals of the X-ray wave field *in search* (1) over *z*_1_-coordinate, namely^17^:

11 $$\begin{aligned} & A_{{\text{R}}} \left( {{\mathbf{q}},{\mathbf{q}}_{0} } \right) = - \frac{{\chi k^{2} }}{4\pi }\int {d^{2} {\mathbf{x}}}_{1} e^{{ - i{\mathbf{q}} \cdot {\mathbf{x}}_{1} }} \int\limits_{\rm T}^{{h\left( {\mathbf{x}} \right)}} {dz_{1} y_{1} \left( {z_{1} ,q} \right)E\left( {{\mathbf{x}}_{1} ,z_{1} } \right)} \hfill \\ , & A_{{\text{T}}} \left( {{\mathbf{q}},{\mathbf{q}}_{0} } \right) = - \frac{{\chi k^{2} }}{4\pi }\int {d^{2} {\mathbf{x}}}_{1} e^{{ - i{\mathbf{q}} \cdot {\mathbf{x}}_{1} }} \int\limits_{\rm T}^{{h\left( {\mathbf{x}} \right)}} {dz_{1} y_{2} \left( {z_{1} ,q} \right)E\left( {{\mathbf{x}}{}_{1},z_{1} } \right)} \hfill \\ \end{aligned}$$

To calculate the scattering amplitude $A_{{\text{R}}} \left( {q,q_{0} } \right)$, taking into account the specific z-coordinate behavior features of the X-ray wave field *in search* (1) and the $y_{1} \left( {z_{1} ,q} \right)$-Eigenfunction (see the first-line Eq. (6)), the low limit of integral over *z*_1_-coordinate *T* it is convenient to be negative and value of |*T*|>> the r.m.s. roughness *σ*. To be specific, the last condition is needed to exclude *T*-interfering effects due to high-valued roughness field *h*(**x**) (remind that the *T* is nothing else the auxiliary parameter being called up to overcome math complexities of working in the framework of the standard Green function formalism^17^, where parameter *T* seems to be equal to zero.

Then, combining the first-line Eqs. (10)–(11) and using the similar math-calculation scheme^17^, one obtains the integral equation to determine the reflected wave amplitude *B*(**q**), namely:

12 $$\begin{aligned} & \frac{1}{{\left( {2\pi } \right)^{2} }}\int {\frac{{d^{2} {\mathbf{q}}_{1} B( {{\mathbf{q}}_{1} } )}}{{( {k_{z} ( {q_{1} } ) - \kappa_{z} ( q )} )}}} \int {d^{2} {\mathbf{x}}} e^{{ - i( {{\mathbf{q}} - {\mathbf{q}}_{1} } ) \cdot {\mathbf{x}} + i\left( {\kappa_{z} \left( q \right) - ik_{z} \left( {q_{1} } \right)} \right)h\left( {\mathbf{x}} \right)}} \hfill \\ &\quad =\frac{1}{{\left( {\kappa_{z} \left( q \right) + k_{z} } \right)}}\int {d^{2} {\mathbf{x}}} e^{{ - i\left( {{\mathbf{q}} - {\mathbf{q}}_{0} } \right) \cdot {\mathbf{x}} + i\left( {\kappa_{z} \left( q \right) + k_{z} } \right)h\left( {\mathbf{x}} \right)}} . \hfill \\ \end{aligned}$$

It is seen that the Eq. (12) does not contain the auxiliary parameter *T* and provides the reflected wave amplitude *B*(**q**) in the form of integral self-consistent integral relationship, not linking with the transmitted wave amplitude *C*(**q**) in opposite to the standard Green function theory^17^, in which they form a system of the two integral equations linked each to other.

Similar to the above case, combining the second-line Eqs. (10), (11) and following the standard math scheme developed in^17^, one obtains the integral relationship for the transmitted wave amplitude *C*(**q**) can be derived in the form

13 $$\begin{aligned} & \frac{1}{{\left( {2\pi } \right)^{2} }}\int {d^{2} {\mathbf{q}}}_{1} C\left( {{\mathbf{q}}_{1} } \right)\int {d^{2} {\mathbf{x}}} e^{{ - i\left( {{\mathbf{q}} - {\mathbf{q}}_{1} } \right) \cdot {\mathbf{x}}}} \frac{{e^{{i\left( {\kappa_{z} \left( {q_{1} } \right) - k_{z} \left( q \right)} \right)h\left( {\mathbf{x}} \right)}} }}{{( {\kappa_{z} ( {q_{1} } ) - k_{z} ( q)} )}} \\ &\quad = \frac{{{\text{T}}_{1} }}{{\left( {\kappa_{z} - k_{z} } \right)}}\left( {2\pi } \right)^{2} \delta_{2} \left( {{\mathbf{q}} - {\mathbf{q}}_{0} } \right), \hfill \\ \end{aligned}$$

provided that the auxiliary parameters *T* needs to be chosen positive, *T* >> *σ* (evidently, Eq. (13) does not contain parameter *T* ).

In general, self-consistent (non-averaged) wave field Eqs. (12), (13) allow to developing the relatively simple procedure for solving the GISAXS issue of the **q**-Eigenwaves propagating through a twofold rough-surfaced medium. Notice that in the case of the step-like abrupt electric susceptibility χ(***r***) = χΘ(z), when the surface roughness field *h*(**x**) = 0, Eqs. (12), (13) definitely provide the conventional Fresnel solutions for the wave amplitudes *B*(**q**) and *C*(**q**). It is important that both the Eqs. (12) and (13) are free out of auxiliary parameter *T* and form the theoretical background of the GISAXS theory in terms of the **q-**Eigenwaves propagating through the twofold rough-surfaced medium.

Underneath, we will apply the self-consistent Eq. (12) to build up analytical solutions for describing the specular (coherent) and diffuse (incoherent) GISAXS from a randomly rough-surfaced medium.

## Zero- and first-order perturbation theory solutions of the GISAXS problem

To build up the theoretical foundation of the specular and non-specular GISAXS phenomenon from a randomly rough surface, one applies the averaged roughness field approach. Integral Eq. (12) can be identically rewritten as follows

14 $$\begin{aligned} &\frac{1}{{\left( {2\pi } \right)^{2} }}\int {\frac{{d^{2} {\mathbf{q}}_{1} B\left( {{\mathbf{q}}_{1} } \right)}}{{\left( {k_{z} \left( {q_{1} } \right) - \kappa_{z} \left( q \right)} \right)}}} \int {d^{2} {\mathbf{x}}} e^{{ - i\left( {{\mathbf{q}} - {\mathbf{q}}_{1} } \right) \cdot {\mathbf{x}}}} \left[ \begin{array}{ll} W_{ - } \left( {q,q_{1} } \right) + \hfill \\ \left( {e^{{i\left( {\kappa_{z} \left( q \right) - ik_{z} \left( {q_{1} } \right)} \right)h\left( {\mathbf{x}} \right)}} - W_{ - } \left( {q,q_{1} } \right)} \right) \hfill \\ \end{array} \right] \hfill \\ &\quad =\frac{1}{{\kappa_{z} \left( q \right) + k_{z} \left( {q_{0} } \right)}}\int {d^{2} {\mathbf{x}}} e^{{ - i\left( {{\mathbf{q}} - {\mathbf{q}}_{0} } \right) \cdot {\mathbf{x}}}} \left[ \begin{array}{ll} W_{ + } \left( {q,q_{0} } \right) + \hfill \\ \left( {e^{{i\left( {\kappa_{z} \left( q \right) + ik_{z} \left( {q_{0} } \right)} \right)h\left( {\mathbf{x}} \right)}} - W_{ + } \left( {q,q_{0} } \right)} \right) \hfill \\ \end{array} \right]. \hfill \\ \end{aligned}$$

Here, the averaged exponential factors *W*_−_(*q*, *q*_1_), *W*_+_(*q*, *q*_1_) are defined as.

15 $$W_{ - } \left( {q,q_{1} } \right) = \left\langle {e^{{i\left( {\kappa_{z} \left( q \right) - k_{z} \left( {q_{1} } \right)} \right)h\left( {\mathbf{x}} \right)}} } \right\rangle , \quad W_{ + } \left( {q,q_{1} } \right) = \left\langle {e^{{i\left( {\kappa_{z} \left( q \right) + k_{z} \left( {q_{1} } \right)} \right)h\left( {\mathbf{x}} \right)}} } \right\rangle ,$$

where the symbol $\left\langle {\ldots} \right\rangle$ denotes the general ensemble-statistics average procedure.

By using the Gaussian ensemble-statistics, the averages (15) can be analytically calculated, which are nothing else the exponential GISAXS factors

15a $$W_{ - } \left( {q,q_{{1}} } \right) = {\exp}\left[ { - 0.{5}\left( {k_{{\text{z}}} \left( q \right) - k_{{\text{z}}} \left( {q_{{1}} } \right)} \right)^{{2}} s^{{2}} } \right], W_{ + } \left( {q,q_{{1}} } \right) = {\exp}\left[ { - 0.{5}\left( {k_{{\text{z}}} \left( q \right) + k_{{\text{z}}} \left( {q_{{1}} } \right)} \right)^{{2}} s^{{2}} } \right].$$

Confining ourselves by terms of the zero- and first-order perturbation theory for deriving the reflected wave amplitude *B*(**q**), a standard procedure yields the following expressions for the reflected wave amplitude *B*(**q**)

16 $$\begin{aligned} \, &B\left( {\mathbf{q}} \right) \cong B_{0} \left( {\mathbf{q}} \right) + B_{1} \left( {\mathbf{q}} \right), \hfill \\ & B_{0} \left( {\mathbf{q}} \right) = \left( {2\pi } \right)^{2} \delta_{2} \left( {{\mathbf{q}} - {\mathbf{q}}_{0} } \right)R_{1} \left( {q_{0} } \right)f_{{\text{R}}} \left( {q_{0} } \right), \quad f_{{\text{R}}} \left( {q_{0} } \right) = \frac{{W_{ + } \left( {q_{0} \, ,q_{0} } \right)}}{{W_{ - } \left( {q_{0} ,q_{0} } \right)}}, \hfill \\ \end{aligned}$$

17 $$\begin{aligned} & B_{1} \left( {\mathbf{q}} \right)\frac{{W_{ - } \left( {q,q} \right)}}{{\left( {k_{z} \left( q \right) - \kappa_{z} \left( q \right)} \right)}} \hfill \\ &\quad=\frac{{R_{1} \left( {q_{0} } \right)}}{{\left( {\kappa_{z} \left( q \right) - k_{z} \left( {q_{0} } \right)} \right)}}\frac{{W_{ + } \left( {q_{0} ,q_{0} } \right)}}{{W_{ - } \left( {q_{0} ,q_{0} } \right)}}\int {d^{2} {\mathbf{x}}} e^{{ - i\left( {{\mathbf{q}} - {\mathbf{q}}_{0} } \right) \cdot {\mathbf{x}}}} \left( {e^{{i\left( {\kappa_{z} \left( q \right) - ik_{z} \left( {q_{0} } \right)} \right)h\left( {\mathbf{x}} \right)}} - W_{ - } \left( {q,q_{0} } \right)} \right) \hfill \\ &\qquad+\frac{1}{{\left( {k_{z} \left( {q_{0} } \right) + \kappa_{z} \left( q \right)} \right)}}\int {d^{2} {\mathbf{x}}} e^{{ - i\left( {{\mathbf{q}} - {\mathbf{q}}_{0} } \right) \cdot {\mathbf{x}}}} \left( {e^{{i\left( {\kappa_{z} \left( q \right) + ik_{z} \left( {q_{0} } \right)} \right)h\left( {\mathbf{x}} \right)}} - W_{ + } \left( {q,q_{0} } \right)} \right). \hfill \\ \end{aligned}$$

Notice that the corresponding equation for specular wave amplitude (16) obtained is identical to the expression first derived in the work^11^. Accordingly, one can utilize analytical Eqs. (16) and (17) for the zero- and first-order perturbation theory amplitudes *B*_0_(**q**) and *B*_1_(**q**) to determine the 1 D specular and 2D diffuse GISAXS scans.

As a result, expression for the reflected 2D intensity distribution can be written in the form^13,17^

18 $$\begin{aligned} \frac{{dR_{tot} \left( {\theta ,\phi } \right)}}{d\Omega } = \left( {2\pi } \right)^{ - 2} k^{2} S_{2}^{ - 1} \frac{{\sin^{2} (\theta )}}{{\sin (\theta_{0} )}}\left\langle {\left| {B_{0} \left( {\mathbf{q}} \right) + B_{1} \left( {\mathbf{q}} \right)} \right|^{2} } \right\rangle \hfill \\ \end{aligned}$$

and represents by itself the superposition of the two terms, namely:

19 $$\frac{{dR_{tot} \left( {\theta ,\phi } \right)}}{d\Omega } = \frac{{dR_{spe} \left( \theta \right)}}{d\Omega } + \frac{{dR_{dif} \left( {\theta ,\phi } \right)}}{d\Omega }$$

($d\Omega = \cos \theta d\theta d\phi$ is the elementary solid angle for the reflected beam, *S*_2_ is an area of a rough surface impinged on by the incident X-ray beam).

Directly following the math procedure scheme^13,17^, standard calculations yield the analytical equations, which describe the 1D specular intensity distributions

20 $$\begin{aligned} &\frac{{dR_{spec} \left( \theta \right)}}{d\Omega } = k^{2} \sin \theta_{0} \delta_{2} \left( {{\mathbf{q}} - {\mathbf{q}}_{0} } \right)\left| {R_{1} \left( {q_{0} } \right)} \right|^{2} f_{R} \left( {q_{0} } \right), \, \hfill \\ & f_{{\text{R}}} \left( {q_{0} } \right) = {\text{Exp}}\left[ {{ - 4}k_{{\text{z}}} \left( {q_{0} } \right){\text{Re}} \kappa_{z} \left( {q_{0} } \right)\sigma^{2} } \right] \hfill \\ \end{aligned}$$

and the diffuse 2D intensity distribution

21 $$\frac{{dR_{dif} \left( {\theta ,\phi } \right)}}{d\Omega } = \left( {2\pi } \right)^{ - 2} k^{2} \frac{{\sin^{2} \left( \theta \right)}}{{\sin \left( {\theta_{0} } \right)}}\left| {\frac{{\left( {k_{z} \left( q \right) - \kappa_{z} \left( q \right)} \right)}}{{W_{ - } \left( {q,q} \right)}}} \right|^{2} \Phi_{{\text{R}}} \left( {q,q_{0} } \right)PSD_{2D} \left( {\left| {{\mathbf{q}} - {\mathbf{q}}_{0} } \right|} \right),$$

remind that $$\left\langle {B_{1} \left( {\mathbf{q}} \right)} \right\rangle = 0$$.

Then, specular reflectivity *R*_spe_(*θ*_0_) and diffuse scattering intensity (DSI) *dR*_dif_(*θ*)/*dθ* are equal to^17^

22 $$R_{{{\text{spec}}}} \left( {\theta_{0} } \right) = \left| {R_{1} \left( {q_{0} } \right)} \right|^{2} f_{{\text{R}}} \left( {q_{0} } \right),$$

23 $$\frac{{dR_{dif} }}{d\theta }\left( \theta \right) = \left( {2\pi } \right)^{ - 2} \frac{{\cos \left( \theta \right)k_{z}^{2} \left( q \right)}}{{\sin \left( {\theta_{0} } \right)}}\left| {\frac{{\left( {k_{z} \left( q \right) - \kappa_{z} \left( q \right)} \right)}}{{W_{ - } \left( {q,q} \right)}}} \right|^{2} \Phi_{R} \left( {q,q_{0} } \right)PSD_{1D} \left( {\left| {q - q_{0} } \right|} \right)$$

The functions $PSD_{2D} \left( {\left| {{\mathbf{q}} - {\mathbf{q}}_{0} } \right|} \right)$ and $PSD_{1D} \left( {\left| {q - q_{0} } \right|} \right)$ and expressions for specular factor $f_{{\text{R}}} \left( {q_{0} } \right)$ and non-specular scattering factor $\Phi_{{\text{R}}} \left( {q,q_{0} } \right)$, are derived as described in Sections A and B of Supplemental Material (cf.^13,15–21^).

Thus, the resulting analytical Eqs. (20)–(23) provide the GISAXS issue solution in a framework of the modified Green function formalism and in terms of the assumed Gaussian ensemble-statistics model for a randomly oriented fractal surface^17–20^. Noteworthy is the fact that according to our calculations, the specular scattering factor *f*_R_(*q*_0_), Eq. (20), is identical to Nevot-Croce’s one^11^. Staying in the scope of the adopted surface model and the first-order perturbation theory used to solving self-consistent (non-averaged) wave field Eq. (12), one can state that expressions (20)–(23) provide in the proper way analytical description of the GISAXS issue and allow to evaluate the 1D specular *R*_spec_(*θ*_0_) and 2D non-specular $\frac{{dR_{dif} \left( {\theta ,\phi } \right)}}{d\Omega }$ scans, and the 1D DSI $\frac{{dR_{dif} \left( \theta \right)}}{d\theta }$ scan as well.

Before proceeding further, it remains to see how well the developed approach works, its validity range, and prospects of solution of the GIAXS issue taking into account the high-order scattering effects. Now, one thing is clear that consideration of the GISAXS issue in the frame of high-order perturbation theory based on Kubo’s cumulant average diagram technique (see, e.g.,^22^). By using such the technique, the self-consistent (non-averaged) wave field Eq. (12) can be converted to the Dyson-type and Bethe–Salpeter-type integral equations to describing the specular and non-specular GISAXS scattering, respectively. In this respect, by using the Kubo technique^22^, endeavor has been undertaken in the work^23^, in which the Dyson-type equation has been derived and analyzed in application to the specular GISAXS scattering. As was shown in^23^, specular scattering factor *f*_R_(*q*_0_) becomes dependent on both the r.m.s. roughness σ and roughness correlation length *ℓ*. Clearly, application of the Kubo technique could enable to improving the present GISAXS theory approach clarifying at least, its validity range.

In general, real surface does not satisfy to the Gaussian ensemble-statistics and *K*_2_-correlation fractal surface model adopted in the present study. As was pointed out in^13–17^, such the model has been applied to clarify the GISAXS peculiar pattern. Here, we can only state that the modified Green function formalism does work and could be applied in a general case of the surface model provided that the ad hoc model of a rough surface is in a matter. For instance, instead of the play-in model, one can utilize the surface model, in which χ(**r**) = 0 for z < 0 whereas for z > 0 and χ(**r**) =  < χ(z) >  + δχ(**r**) at each point **r** = **( x,** z**)** of the statistically distorted surface layer, where δχ(**r**) is the fluctuating part of χ(**r**), < δχ(**r**) >  = 0.

## Numerical run-through for providing the GISAXS patterns: results and discussion

In this section, based on formulae (20)**–**(23), the numerically simulated results for the 1D specular and 1D-2D non-specular GISAXS patterns from a randomly rough fractal surface based are presented. In particular, one presents the results of proper calculations for different dimensionless values of fractal surface parameters as *kσ* and $k\ell$ and the FSMI *h* as well.

**Figure 2:**
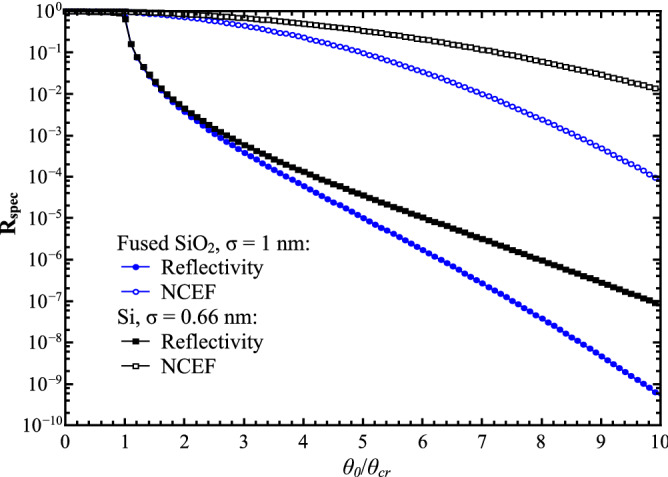
Simulated 1D specular scans $$R_{spec}$$ *versus* the normalized grazing-incidence angle $${\raise0.7ex\hbox{${\theta_{0} }$} \!\mathord{\left/ {\vphantom {{\theta_{0} } {\theta_{cr} }}}\right.\kern-\nulldelimiterspace} \!\lower0.7ex\hbox{${\theta_{cr} }$}}$$. The dimensionless r.m.s. roughness *kσ*: 40.7999 (fused quartz—blue color), 26.9279 (Si(111)—black color). *CuK*α_1_-radiation, λ = 0.154 nm.

The numerically simulated 1D scans *R*_spec_(*θ*_0_) *versus* the grazing-incidence angle *θ*_0_ in the angular range ${0} \le \theta_{{0}} \, \le { 10}\theta_{{{\text{cr}}}} \,$ are shown in Fig. 2 in the cases of the fused quartz and Si(111) samples. The corresponding parameters *kσ* are equal to 40.7999 (*σ* = 1 nm for fused quartz) and 26.9279 (*σ* = 0.66 nm for Si(111)). Simultaneously, in Fig. 2 the curves of Nevot-Croce’s exponential factors (NCEFs) are depicted. It is evidently seen that the curve NCEF for the fused quartz (blue curve) goes below to the curve for Si(111) (black curve) due to the about two-times difference in their r.m.s. σ values.

**Figure 3:**
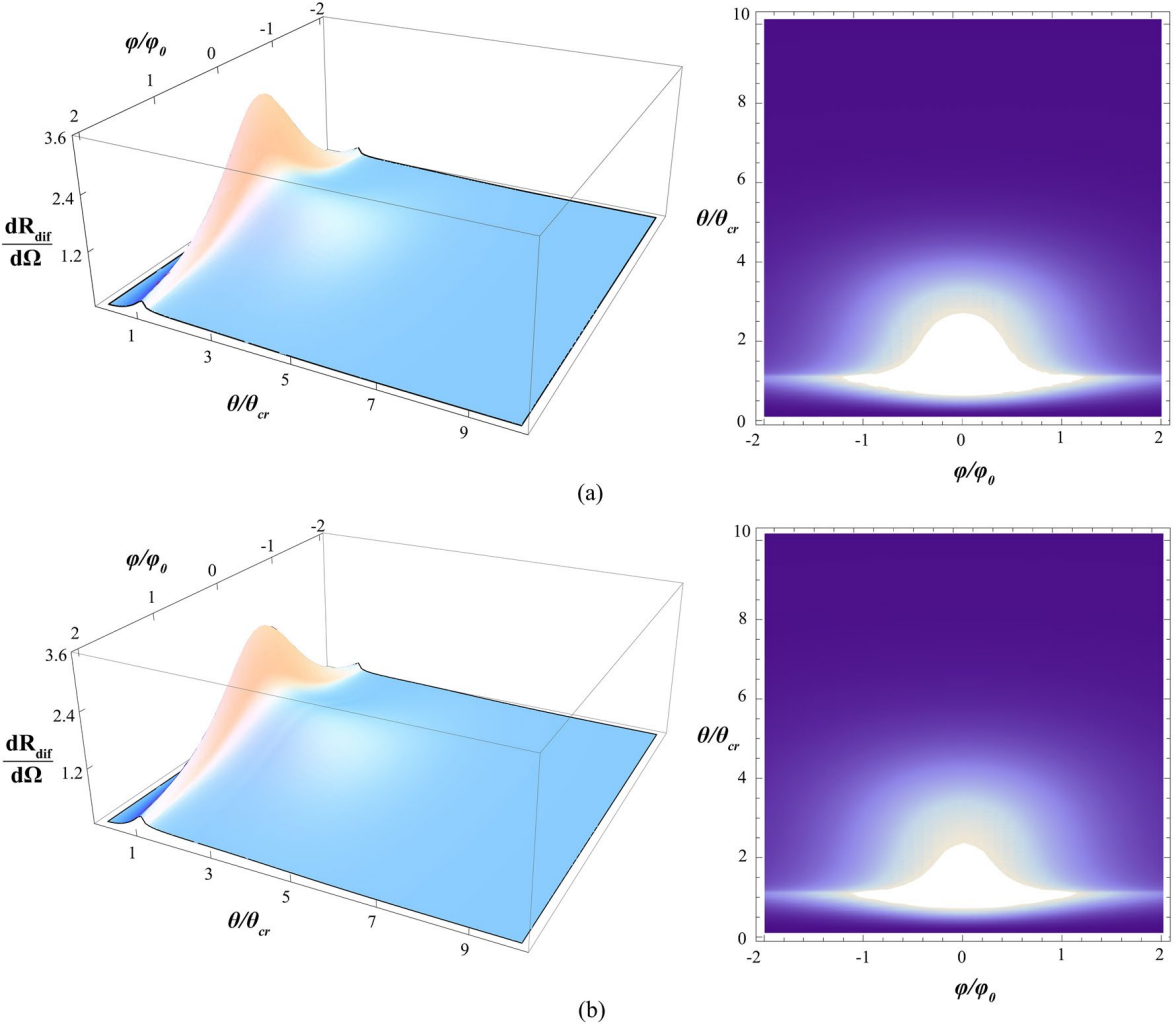
Simulated 3D- and 2D-plots of the normalized non-specular scan $${\raise0.7ex\hbox{${\frac{{dR_{dif} \left( {\theta ,\phi } \right)}}{d\Omega }}$} \!\mathord{\left/ {\vphantom {{\frac{{dR_{dif} \left( {\theta ,\phi } \right)}}{d\Omega }} {\frac{{dR_{dif} \left( {\theta_{0} ,\phi = 0} \right)}}{d\Omega }}}}\right.\kern-\nulldelimiterspace} \!\lower0.7ex\hbox{${\frac{{dR_{dif} \left( {\theta_{0} ,\phi = 0} \right)}}{d\Omega }}$}}$$. The grazing-incidence angle *θ*_0_ = 2*θ*_cr_. The correlation length $$\ell$$ = 0.2451 μm. The scattering polar *θ* and azimuth $$\phi$$ angles are measured in units of the critical angle *θ*_cr_ and *φ*_0_ = $$\left( {k\ell } \right)^{ - 1}$$. The FSMI *h* = 1/2. The dimensionless r.m.s. roughness *k*σ: 40.7999, the fused quartz (**a**); 26.9279, Si(111) (**b**).

**Figure 4:**
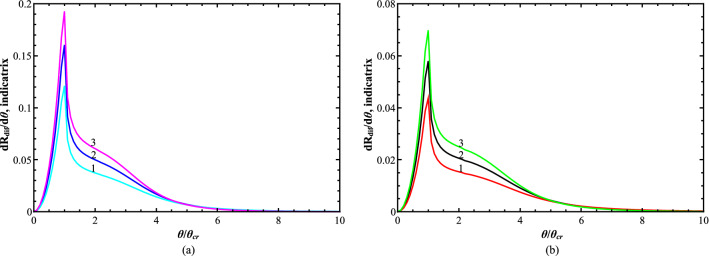
Simulated 1D DSI $\frac{dR}{{d\theta }}\left( \theta \right)$-scans. The grazing-incident angle *θ*_0_ = 2*θ*_cr_. The FSMI *h* = {1/3, 1/2, 2/3}. The correlation length $\ell$ = 0.2451 μm. The dimensionless r.m.s. roughness *k*σ: = 40.7999, the fused quartz (**a**); 26.9279, Si(111) (**b**).

Some examples of the 2D non-specular intensity distribution $\frac{{dR_{dif} \left( {\theta ,\phi } \right)}}{d\Omega }$ and 1D DSI $\frac{{dR_{dif} \left( \theta \right)}}{d\theta }$ numerically simulated according to analytical formulae (21) and (23) are presented in Figs. 3a, 4a for the fused quartz and in Figs. 3b, 4b for Si(111), respectively.

As it follows from Eqs. (21), (23), they contain the non-specular scattering factor $\Phi_{R} \left( {q,q_{0} } \right)$, the explicit expression of which is done in Section B of Supplemental Material (cf.^15–18^). It represents by itself the superposition of the exponential factors, which contain the r.m.s. roughness *σ* to be squared. Dependence of the 1D $\frac{{dR_{dif} \left( \theta \right)}}{d\theta }$—and 2D $\frac{{dR_{dif} \left( {\theta ,\phi } \right)}}{d\Omega }$-scans on the correlation length $\ell$ and the FSMI parameter *h* are due to the *PSD*_1D_(|*q*-*q*_0_|)- and *PSD*_2D_(|**q**-**q**_0_|)-functions, the corresponding expressions of which are given in Section A of Supplemental Material (cf.^17^).

Simulated scans displaced in Figs. 3, 4 have been calculated for the *CuK*_α1_ X-ray radiation. The corresponding wavelength and correlation length $\ell$ are assumed to be 0.154 nm and 0.2451 μm. The grazing-incidence angle *θ*_0_ is chosen to be 2*θ*_cr_. The r.m.s. roughness *σ* is assumed to be 1 nm (fused quartz in Figs. 3a, 4a) and 0.66 nm (Si(111) in Figs. 3b, 4b), respectively; the FSMI *h* = 1/2 (Fig. 3) and *h* = {1/3, 1/2, 2/3} (Fig. 4). In general, for the fused quartz and Si(111), the calculated 2D $\frac{{dR_{dif} \left( {\theta ,\phi } \right)}}{d\Omega }$- and 1D DSI $\frac{{dR_{dif} \left( \theta \right)}}{d\theta }$-scans for the fused quartz and Si(111) are topologically similar. At the same time, they differ in their maximum values by about half. Physically, it can be explained that for the fused quartz, Figs. 3a, 4a, the diffuse GISAXS is about two-times stronger than it is for Si(111), Figs. 3b, 4b, due to the two-times difference in values of the corresponding r.m.s. roughness σ.

Following to^17^, in parallel to the grazing-incidence angle *θ*_0 _= 2*θ*_cr_, the corresponding 2D $\frac{{dR_{dif} \left( {\theta ,\phi } \right)}}{d\Omega }$—and 1D $\frac{{dR_{dif} \left( \theta \right)}}{d\theta }$-scans have been evaluated for the grazing-incidence angle *θ*_0 _= 3*θ*_cr_.

Direct comparison of the scans calculated for *θ*_0 _= 2*θ*_cr_ and *θ*_0 _= 3*θ*_cr_ respectively, support conclusion earlier made^17^ that the 2D $\frac{{dR_{dif} \left( {\theta ,\phi } \right)}}{d\Omega }$- and 1D $\frac{{dR_{dif} \left( \theta \right)}}{d\theta }$-scans reveal Yoneda’s scattering peak^24^ at the scattering angle *θ* = *θ*_cr_^23^ and support statement that Yoneda’s scattering peak decreases (increases) in respect to the mirror scattering peak at *θ* = *θ*_0_, *θ*_0_ > *θ*_cr_, with decreasing (increasing) the grazing-incidence angle *θ*_0_.

Further, we will define the exponential Nevot-Croce’s function as *NCF*(*q*_0_) $\equiv$ − 2ln|*f*_R_(q_0_)| and the *DSF*(*q*, *q*_0_)-function as a logarithm of the *PSD*_1D_(|*q*-*q*_0_|)-function extracted from Eqs. (20), (23) (cf. equation (A. 5) for the *PSD*_1D_(|*q*-*q*_0_|)-function), namely:

24 $$NCF\left( {q_{0} } \right) = [k_{{\text{z}}}^{{2}} \left( {q_{0} } \right) + {\text{Re}}(k_{{\text{z}}}^{{2}} \left( {q_{0} } \right)) + {2}k_{{\text{z}}} \left( {q_{0} } \right){\text{Re}}(k_{{\text{z}}} \left( {q_{0} } \right))]s^{{2}}$$

25 $$DSF\left( {\left| {q - q_{0} } \right|} \right) = \ln \left[ {\frac{{k^{2} \ell }}{{\left( {qq_{0} } \right)^{1/2} \left( {1 + \left( {k\ell } \right)^{2} \left( {q - q_{0} } \right)^{2} } \right)^{1/2 + h} }}} \right]$$

Notice that the above functions *NCF*(*q*_0_) and *DSF*(*q *− *q*_0_) depend on the model parameters {*σ*, *ℓ*, *h*} that altogether characterize the Mandelbrot’s rough fractal surface. Extracting functions (24), (25) from the experimental GISAXS data provides the retrieval of these Mandelbrot’s parameters by using the standard least-squared procedure in a *χ*^2^-fit fashion^25^. As was pointed out in^17^, the best way for that is to use the asymptotical 1D *R*_spec_(*q*_0_)- and 1D DSI(*q*, q_0_)-curves for the scattering angles *θ* to be much more than the mirror scattering angle *θ*_0_, *θ*_0_ > *θ*_cr_.

**Figure 5:**
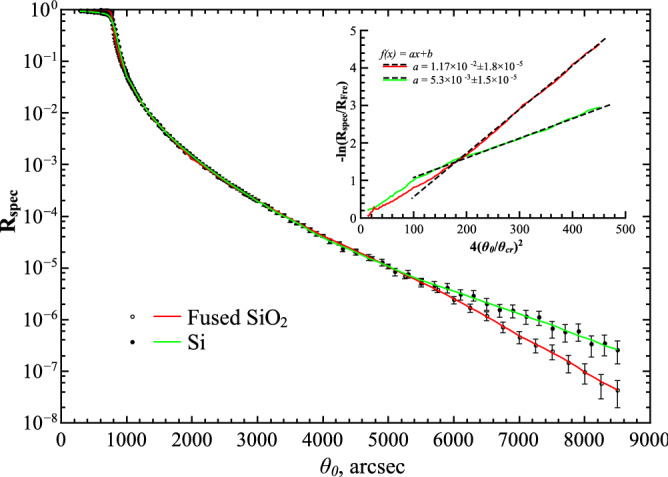
Experimental 1D $R_{spec} \left( {\theta_{0} } \right)$-curves versus the grazing-incidence angle *θ*_0_, and functions NCF(*θ*_0_) = $- \ln \left[ {R_{spec} \left( {\theta_{0} } \right)/R_{Fre} \left( {\theta_{0} } \right)} \right]$ *versus* the grazing-incidence angle squared 4(*θ*_0_/*θ*_cr_)^2^. Functions NCF: the fused quartz (red color) and Si(111) (green) are given in the insert. The r.m.s. roughness *σ*, which match the experimental curves $- \ln \left[ {R_{spec} \left( {\theta_{0} } \right)/R_{Fre} \left( {\theta_{0} } \right)} \right]$ are equal to: 0.702753 ± 0.357332 × 10^–3^ nm (the fused quartz) and 0.471035 ± 0.652408 × 10^–3^ nm (Si(111)).

Hereafter, having aim to demonstrate how it works, retrieval of the r.m.s. parameter *σ* from the 1D experimental *R*_spec_(*q*_0_) data for the fused quartz and Si(111) samples that are displayed in Fig. 5.

Both the sample surfaces have been finished by mechanical polishing with iron oxide powders, and then, have been cleaned with ethanol just before the experimental measurements. The extracted *NCF*-functions *versus* the variable 4(*θ*_0_/ *θ*_cr_)^2^ are displayed for interval of the grazing-incidence angles 5 *θ*_cr_ ≤ *θ*_0_ ≤ 12.5 *θ*_cr_ in the insert in Fig. 5.

At last, by using a direct least -squared procedure in a χ^2^-fit fashion^25^, the values of the r.m.s. roughness *σ* have been obtained. Accordingly, they are equal to: 0.702753 ± 0.357332 × 10^–3^ nm (fused quartz) and 0.471035 ± 0.652408 × 10^–3^ nm (Si(111)) samples.

## Concluding remarks

In conclusion, we can state that the current theoretical approach in the framework of the modified Green function formalism provides a plausible treatment of the GISAXS issue beyond the scope of the conventional DWBA^13^ and preceding author’s theory^17^. Unlike the GISAXS theory earlier developed in^17^, the modified Green function formalism provides novel self-consistent (non-averaged) wave field Eqs. (12) and (13) for the reflected and transmitted **q**-Eigenwave amplitudes *B*(**q**) and *C*(**q**), respectively. Based on the Gaussian ensemble-statistics averaging procedure of random roughness field *h*(**x**), the integral Eq. (12) for the reflected wave amplitude *B*(**q**) has been exploited to search the zero- and first-order perturbation theory solutions of the GISAXS issue. These solutions are obtained in the analytical form of expressions (20)–(23) for determining the 1D specular (coherent) and 2D non-specular (incoherent) intensity distributions in the case of the GISAXS by a twofold rough-surfaced medium. To develop the current GISAXS approach, we have advanced the Green function formalism and, besides, some key assumptions have been assumed. Namely, the Gaussian ensemble-statistics and Mandelbrot’s fractal surface model have been exploited. Appropriately, the specular *f*_R_(*q*_0_) and non-specular $\Phi_{R} \left( {q,q_{0} } \right)$ scattering have been derived. The specular scattering factor *f*_R_(*q*_0_) identically coincides with the corresponding expression earlier obtained in^11^, whereas in the case of the non-specular GISAXS, new expressions (20)–(23) for describing the 1D and 2D GISAXS intensity distributions have been derived in terms of the *PSD*_2D_(|*q *− *q*_0_|)- and *PSD*_1D_(|*q *− *q*_0_|)-functions, respectively.

At last, based on formulae (24), (25), the inverse issue of retrieving parameters {σ, *ℓ*, *h*} of Mandelbrot’s rough surface model from the experimental GISAXS data has been shortly discussed. Accordingly, applying a direct fit-procedure in a χ^2^-sense^25^ and using the theoretical representation of function *NCF* (24) in the case of the experimental specular GISAXS data collected for the fused quartz and Si(111) samples, retrieving the r.m.s. roughness parameter *σ* has been successfully realized (see Fig. 5).

## Supplementary information

Supplementary Information.

## Data Availability

The raw data required to reproduce these findings are available upon request.
