# Supplementary material for: Novel theoretical approach to the GISAXS issue: the Green function formalism using the q-Eigenwaves propagating through a twofold rough-surfaced medium
Source: Sci Rep. 2020 Jul 14;10:11547. doi: 10.1038/s41598-020-68326-2 (PMC7360765; doi:10.1038/s41598-020-68326-2)
Supplement: Supplementary file 1 — Supplementary Information. [file 41598_2020_68326_MOESM1_ESM.docx]

**Supplement to “Novel theoretical approach to the GISAXS issue:**

**the Green function formalism using the *q*-Eigenwaves propagating through a twofold rough-surfaced medium”**

**F. N. Chukhovskii and B.S. Roshchin**

*Shubnikov Institute of Crystallography of Federal Scientific Research Centre "Crystallography and photonics", Russian Academy of Sciences, Leninsky pr. 59, Moscow, 119333, Russia*

**Section A. Power spectral density functions**

According to ^17,18^, the two-point cumulant correlation function K_2_(*x*),$x=\left(\left|\mathbf{x}_{1}-\mathbf{x}_{2}\right|\right)$, is equal to unity for $x=0$ and tends to zero for , the lateral 2D vector $\mathbf{x}=\mathbf{x}_{1}-\mathbf{x}_{2}$ is lying within the reference plane *z*=0, is the correlation length.

In order to derive the power spectrum density functions, $\text{PSD}_{2D,1D}\left(q\right)$, involved into the formulae (21), (23), which determine the 2D $\frac{dR}{d\Omega}\left(\theta,\phi\right)$ - and the 1D DSI $\frac{dR}{d\theta}\left(\theta\right)$ - scans, respectively. For that, one needs first to go on over the correlation function K_2_(*x*) to the function $\text{PSD}_{2D}\left(\left|\mathbf{q}-\mathbf{q}_{0}\right|\right)$ that is nothing else the 2D inverse (**q**-**q**_0_)-Fourier transform of the correlation function K_2_(*x*), where the lateral 2D vectors **q**, **q**_0_ are parallel to the reference plane *z* = 0, $q=\left|\mathbf{q}\right|$, $q_{0}=\left|\mathbf{q}_{0}\right|$ [see Fig. 1].

Following to ^17^, we choose the correlation function K_2_(*x*) in the form proposed in ^19^

.

|  | (A.1) |
| --- | --- |

whereis the modified Bessel function of the second kind, ℓ is the correlation length, *h* is the FSMI index of the Mandelbrot’s fractal model surface, Γ(*h*+1) is the gamma function, 0 < *h* <1 (see ^17, 21^ for details).

Correspondingly,$\text{K}_{2}\left(0\right)=1$ for $x=0$ and for it exponentially tends to zero as $\text{(}h\neq\text{1/2)}$

|  | (A.2) |
| --- | --- |

It is interesting that when the FCMI *h* = ½, the correlation function (A.2) exactly coincides with the exponential function for the two-point height-height function ^13^.

Accordingly, taking into account table-valued integrals containing, in particular, a product of the Bessel function $J_{0}\left(\left|\mathbf{q}-\mathbf{q}_{0}\right|x\right)$ and function (A.1) under the integral symbol over the variable *x*, the $\text{PSD}_{2D}\left(\left|\mathbf{q}-\mathbf{q}_{0}\right|\right)$-function can be cast in the form ^17, 21^

| , | (A.3) |
| --- | --- |

and respecting to expression for $\text{PSD}_{1D}\left(q,q_{0}\right)$ defined as the integral of the $\text{PSD}_{2D}\left(\left|\mathbf{q}-\mathbf{q}_{0}\right|\right)$ function over the azimuth angle *φ*, *i.e.*

| $\text{PSD}_{1D}\left(q,q_{0}\right)=\int_{-\pi}^{\pi} d\phi\text{PSD}_{2D}\left(\left\vert\mathbf{q}-\mathbf{q}_{0}\right\vert\right)$, | (A.4) |
| --- | --- |

in the limit of the dimensionless correlation length parameter *kℓ* to be much more than unity, direct math calculations yield the ultimate expression for integral (A.4) as follows

| , $\Gamma\left(\frac{1}{2}\right)=\left(\pi\right)^{1/2}$. | (A.5) |
| --- | --- |

In the above, the 2D lateral vectors **q** and **q**_0_ are defined: **q** = *k*(cos*θ*cos*φ*, cos*θ*sin*φ*, 0), *q*=*k*cos*θ* and **q**_0_ =*k* (cos*θ*_0_, 0, 0), *q*_0_ = *k*cos*θ*_0_ (*cf*. Fig. 1).

Finally, in the terms of angular variables (*θ*, *θ*_0_; φ), the $\text{PSD}_{2D}\left(\left|\mathbf{q}-\mathbf{q}_{0}\right|\right)$-function can be written in the form ^17^

|  | (A.6) |
| --- | --- |

that seems to be suitable for numerical simulations of the 2D non-specular $\frac{dR}{d\Omega}\left(\theta,\phi\right)$ scans.

**Section B. Non-specular scattering factor**

By using equation (17) for the first-order perturbation theory solution $B_{1}\left(\mathbf{q}\right)$ and taking into account equation $\left\langle B_{1}\left(\mathbf{q}\right)\right\rangle=0$ that is valid in the scope of any ensemble-statistics for the random roughness field *h*(**x**), for evaluating Kato’s average$\left\langle B_{1}\left(\mathbf{q}\right)B_{1}^{*}\left(\mathbf{q}\right)\right\rangle_{cum}$, the standard Gaussian average technique can be applied ^13, 15-22^.

To be specific, as it follows from equations (16), (17) for the zero- and first-order perturbation theory terms *B*_0_(**q**), *B*_1_(**q**), the corresponding averaged expressions involved into the non-specular scattering factor $\Phi_{\text{R}}\left(q,q_{0}\right)$ can be written in the form ($\left\langle B_{1}\left(\mathbf{q}\right)\right\rangle=0$, *cf*. ^15-22^ )

|  | (B.1) |
| --- | --- |

where the length-scale scattering factors $\Lambda{}_{+}\left(q,q_{0}\right)$ and $\Lambda{}_{-}\left(q,q_{0}\right)$ are equal to

| . | (B.2) |
| --- | --- |

Accordingly, in the case of the Gaussian ensemble-statistics they are nothing else the exponential factors, namely:

| , | (B.3) |
| --- | --- |
| , | (B.4) |
|  |  |
| , | (B.5) |
| , | (B.6) |
| , | (B.7) |
| . | (B.8) |

It is worth to note that the Gaussian averaged expressions (B.3)-(B.8) have been used to numerical simulations of the 1D specular and 2D non-specular GISAXS intensity distributions according to equations (20)-(23) of main manuscript.

As one concerns to a general case of *ad hoc* roughness-ensemble-statistics to evaluate the non-specular statistical factor$\Phi_{\text{R}}\left(q,q_{0}\right)$, the relevant statistics surface model has to be applied. At this stage, we can only state that the modified Green function theory approach is valid and does work independently on somehow statistical model of a rough fractal surface.
